# Development of a model of Saint Louis encephalitis infection and disease in mice

**DOI:** 10.1186/s12974-017-0837-2

**Published:** 2017-03-22

**Authors:** Rafael Elias Marques, Juliana L. Del Sarto, Rebeca P. F. Rocha, Giovanni F. Gomes, Allysson Cramer, Milene A. Rachid, Danielle G. Souza, Maurício L. Nogueira, Mauro M. Teixeira

**Affiliations:** 1 Immunopharmacology, Departamento de Bioquímica e Imunologia, Instituto de Ciências Biológicas, Universidade Federal de Minas Gerais, Belo Horizonte, Minas Gerais Brazil; 2 Laboratório de Investigação em Neurodegeneração e Infecção, Hospital Universitário João de Barros Barreto, Universidade Federal do Pará, Belém, Pará Brazil; 3 Laboratório de Imunorregulação de Doenças Infecciosas, Departamento de Bioquímica e Imunologia, Instituto de Ciências Biológicas, Universidade Federal de Minas Gerais, Belo Horizonte, Minas Gerais Brazil; 4 Laboratório de Apoptose, Departamento de Patologia Geral, Instituto de Ciências Biológicas, Universidade Federal de Minas Gerais, Belo Horizonte, Minas Gerais Brazil; 5 Laboratório de Interação Microrganismo-Hospedeiro, Departamento de Microbiologia, Instituto de Ciências Biológicas, Universidade Federal de Minas Gerais, Belo Horizonte, Minas Gerais Brazil; 6 Laboratório de Pesquisas em Virologia, Departamento de Doenças dermatológicas, Infecciosas e Parasitárias, Faculdade de Medicina de São José do Rio Preto, São José do Rio Preto, São Paulo Brazil; 7 Present address: Laboratório Nacional de Biociências, Centro Nacional de Pesquisa em Energia e Materiais, Campinas, São Paulo Brazil

**Keywords:** St. Louis encephalitis, SLEV, Mouse model, Viral encephalitis, Inflammation

## Abstract

**Background:**

Flaviviruses are a genre of closely related viral pathogens which emerged in the last decades in Brazil and in the world. Saint (St.) Louis encephalitis virus (SLEV) is a neglected flavivirus that can cause a severe neurological disease that may lead to death or sequelae. St. Louis encephalitis pathogenesis is poorly understood, which hinders the development of specific treatment or vaccine.

**Methods:**

To address this problem, we developed a model of SLEV infection in mice to study mechanisms involved in the pathogenesis of severe disease. The model consists in the intracranial inoculation of the SLEV strain BeH 355964, a strain isolated from a symptomatic human patient in Brazil, in adult immunocompetent mice.

**Results:**

Inoculated mice presented SLEV replication in the brain, accompanied by tissue damage, disease signs, and mortality approximately 7 days post infection. Infection was characterized by the production of proinflammatory cytokines and interferons and by leukocyte recruitment to the brain, composed mainly by neutrophils and lymphocytes. In vitro experiments indicated that SLEV is able to replicate in both neurons and glia and caused neuronal death and cytokine production, respectively.

**Conclusions:**

Altogether, intracranial SLEV infection leads to meningoencephalitis in mice, recapitulating several aspects of St. Louis encephalitis in humans. Our study indicates that the central nervous system (CNS) inflammation is a major component of SLEV-induced disease. This model may be useful to identify mechanisms of disease pathogenesis or resistance to SLEV infection.

**Electronic supplementary material:**

The online version of this article (doi:10.1186/s12974-017-0837-2) contains supplementary material, which is available to authorized users.

## Background

St. Louis encephalitis virus (SLEV) is a causative agent of encephalitis in humans and horses in the Americas [1–3]. SLEV is a member of the *Flavivirus* genus, together with important human pathogens, such as Dengue and Zika viruses, but belongs to the Japanese encephalitis virus (JEV) serocomplex [4]. Like all flaviviruses, SLEV is a single-stranded positive-sense RNA virus, with a genome of approximately 11 kb that encodes three structural genes and seven non-structural genes [5]. SLEV transmission cycles involve Culex mosquitoes, birds, and a variety of mammals [3], although alternative cycles involving other mosquito species have been reported [6]. SLEV is closely related to JEV and West Nile virus (WNV), both also characterized by the ability to cause severe neurological disease in humans [7, 8]. The recent emergence of Zika virus (ZIKV) and the prospect of outbreaks of other flaviviruses indicates how necessary it is to study neglected arboviral diseases [6, 9].

The majority of SLEV infections in humans is asymptomatic or result in flu-like or dengue-like symptoms [10–12]. Severe cases are acute and characterized by intense headache, fever, and neurological alterations such as confusion, convulsions, loss of body reflexes, paralysis, meningitis, and/or encephalitis [13–16]. Mortality rates in severe cases may reach 20%, and survivors often present with neurological sequelae, such as cognitive impairment, memory loss, and incoordination [17, 18]. There are no specific treatments or a vaccine available against St. Louis encephalitis, although prototype vaccines were developed and shown to protect mice against SLEV challenge [19–21].

The development of specific treatments against SLEV infection would benefit from a greater understanding of disease pathogenesis, which is limited and mostly inferred from the study of other flaviviral infections. From this perspective, rodent models represent an important and extensively used tool for the study of flaviviral encephalitis [15, 22, 23]. The earliest reports of experimental SLEV infection in mice were performed in the early 1930s, following SLEV discovery, and consistently described the ability of SLEV to cause brain damage and death [13, 22, 24]. Based on mortality indexes and seroconversion, mouse models of SLEV infection were used to characterize the virulence of SLEV isolates [25], to assess vaccine efficacy [19–21], and to test potential treatments [26, 27]. Moreover, mechanisms leading to SLEV invasion of the central nervous system (CNS), a critical step in the pathogenesis of viral encephalitis, were studied using a hamster model of infection [28]. Overall, factors such as age, route of inoculation, and the viral strain are determinant for mouse and hamster susceptibility to SLEV infection [29].

In this manuscript, we describe a robust model of SLEV infection in C57BL/6J and Balb/c mice that recapitulates several aspects of human disease [13]. This experimental model is based on the intracranial inoculation of a SLEV strain isolated from a symptomatic patient in Brazil [5, 30] into adult immunocompetent mice, which develop severe neurological disease. We found that SLEV replicates in the brains of infected mice, causing the production of proinflammatory cytokines and the recruitment and activation of leukocytes, which is consistent with meningoencephalitis. SLEV infection causes significant brain damage and results in death. Importantly, this study indicates that CNS inflammation is a major component of SLEV-induced disease.

## Methods

### Mice

Eight- to 12-week-old wild type (WT) C57BL/6 or Balb/c mice were purchased from Centro de Bioterismo of UFMG (Belo Horizonte, Brazil). All animals were kept in the laboratory animal facility under controlled temperature (23 °C) with a strict 12-h light/dark cycle, food, and water available ad libitum. All experimental procedures were approved by and complied with the regulations of Universidade Federal de Minas Gerais (UFMG) Committee for Ethics in Animal Use (CEUA), under protocol number 349/2012.

### Virus

SLEV strain BeH 355964 was provided by Prof. Luis Tadeu Moraes Figueiredo (Universidade de São Paulo, SP, Brazil). BeH 355964 stocks were generated by passage in C6/36 mosquito cell monolayers, cultivated in Leibovitz-15 supplemented with 10% *v*/*v* fetal bovine serum (FBS) (Cultilab, Brazil) and antibiotics. Clarified supernatants containing virus were titrated by plaque assay in Vero cells, and viral titers were expressed in plaque forming units (PFU)/mL of supernatant. SLEV BeH 355964 complete genome sequence is available at GenBank under accession number KM267635 [5].

### In vivo experimental infection

Mice were inoculated intracranially (i.c.) or intraperitoneally (i.p.) with different inocula of SLEV or saline, as mock-infected control. Inocula were prepared by diluting viral stocks (as L-15 clarified supernatants) in saline. Viral dilutions in saline were at least 1000-fold, resulting in a solution that contained negligible amounts of the original L-15 culture supernatant. Injected volumes were 20 and 100 μL for i.c. and i.p. routes, respectively. For the i.c. inoculation, mice were anesthetized using Isoflurane (Biochimico, Brazil) 5% *v*/*v* inhalation. A syringe containing the inoculum was positioned perpendicularly to the head on the intersections of medial and sagittal planes, following insertion of the needle into the cranial cavity, injection and perpendicular removal from within the cranial cavity. Mice were observed twice a day for 14 days or up to determined time points for sample collection, at which mice were anesthetized with ketamine/xylazine (Syntec, Brazil) before collection of blood and organs, or euthanized by CO_2_ inhalation. All tissue samples were stored at −80 °C until analysis. On survival experiments, mice presenting with severe disease signs (such as complete paralysis) were euthanized by CO_2_ inhalation and considered dead in data analysis.

### Quantification of viral load

SLEV load in cell culture and tissue samples was determined by plaque assay and/or reverse transcriptase quantitative PCR (RT-qPCR). Tissue samples were processed into 10% *w*/*v* homogenates in DMEM prior to analysis by both techniques. Briefly, the plaque assay consisted in the serial dilution of samples for adsorption in Vero cell monolayers, for an hour. Samples were removed, following the addition of an overlay media containing 1.5% *w*/*v* carboxymethylcellulose (Synth, SP, Brazil) in 2% fetal bovine serum (FBS) *v*/*v* DMEM. After 7 days, plates were fixed with formaldehyde, washed and stained with methylene blue (Synth, SP, Brazil) 1% *w*/*v*. Results were expressed as plaque forming units (PFU)/mL of supernatant or PFU/100 mg of tissue. For the RT-qPCR reaction, samples were submitted to RNA extraction (QIAamp viral RNA extraction kit, QIAgen) and cDNA synthesis using random primers (Promega) and SuperScript Reverse Transcriptase III (Invitrogen), according to the companies’ specifications. The qPCR reaction was performed in the 7500 Fast platform using SYBR green reagents (Applied Biosystems) and primers targeting SLEV NS5 gene (primer *forward* FG1 TCAAGGAACTCCACACATGAGATGTACT, primer *reverse* nSLE ATTCTTCTCTC AATCTCCGT), as described elsewhere [31]. All PCR reactions were accompanied by a standard curve of the 232 bp NS5 amplicon. Results were expressed as relative number of genome copies of SLEV per sample.

### Quantification of cytokines and chemokines

Concentrations of Interferon (IFN) γ, CCL5, CXCL-1, IL-6, IL-1β, IL-10, IL-17, and TNF-α were quantified in cell culture or processed tissue samples by ELISA (R&D Systems, USA), following the manufacturer instructions. The detection limit of quantitative ELISA was in the range of 4–8 pg/mL or picogram per 100 mg of tissue. Results are expressed as picogram per 100 mg of tissue, picogram per milliliter of supernatant or by absorbance at 490 nm. Type I IFN (IFNα and IFNβ) levels were determined in processed samples by RT-qPCR using specific primers (IDT, USA). Cycle threshold (Ct) values of target genes were normalized to the housekeeping gene 18S and analyzed according to the ΔΔCt method: 2^−ΔCt (sample)−ΔCt (housekeeping)^. Results are expressed as fold increase over the mock-infected WT group.

### Determination of enzymatic activity from leukocytes

Assays to detect the activity of myeloperoxidase (MPO), eosinophil peroxidase (EPO), and *N*-acetil-β-d-glucosaminidase (NAG) were performed in tissue samples as a measure of leukocyte recruitment into target organs. To measure MPO and EPO activity, tissue homogenates were prepared in 1 mL of PBS containing 0.5% hexadecyltrimethyl ammonium bromide (HTAB) and 5 mM EDTA. Saline/Triton X-100 0.1% *v*/*v* was used to process tissues for NAG activity measurement. Test protocols were performed as already described [32]. Briefly, samples were evaluated for their ability to convert the substrates *p*-nitrophenyl-β-glycosamine (for NAG), 3,3′ 5,5′ tetramethylbenzidine (for MPO) or *o*-phenylenediamine (for EPO) to generate a colored solution proportional to the amount of enzyme in the sample. Plates for each assay were read at 405, 450, and 495 nm, respectively, and results expressed as absorbance.

### Hematological parameters

Blood was collected from the brachial plexus of anesthetized mice in heparinized tubes for total and differential leukocyte count, platelet count, measurement of the hematocrit index, and, separately, for serum. Platelets and leukocytes were quantified in an optical microscope (Zeiss ICS Standard 25) using a Neubauer chamber or mounted microscope slides, containing Diff-quik-stained samples (Laboclin, Brazil). The hematocrit index was determined on centrifuged blood samples in heparinized glass capillaries. Results are as counts/mm^3^, per milliliter of blood (leukocytes), or percentage (hematocrit).

### Flow cytometry

Mice were euthanized and perfused with 15 mL of PBS. The brains were collected, homogenized individually, and centrifuged against Percoll gradients (35 and 70%) for separation of leukocytes/microglia. Collected cells were washed, counted, and stained with antibodies against leukocyte surface markers (CD3, CD4, CD8, CD19, CD69, GR-1, F4/80, NK1.1) (all purchased from BD Biosciences). Cells were fixed in buffered 4% *v*/*v* formaldehyde and acquired in a FACS Canto II cytometer (BD Biosciences). Analyzed cell populations included lymphocytes, granulocytes, and macrophages/microglia, initially gated by size/granularity and subsequently by the expression of surface markers, and compared to negative and isotype-stained controls. Data were analyzed using FlowJo (Tree Star), and results expressed as total cell number of positive cells per brain/animal.

### Histopathology

Mice were euthanized and perfused with 15 mL of PBS and 15 mL of buffered 4% *v*/*v* formaldehyde, for the collection of brains. Sections (5 μm thick) were made in a rostral to caudal fashion, mounted and stained in H&E. Sections were then analyzed for signs of brain pathology and inflammation, focusing on the regions of the hippocampus and the cortex/meninges. Images were obtained at ×200–400 magnification using an Olympus BX51 optical microscope equipped with a camera.

### Behavioral assessment

Behavioral changes induced in mice by SLEV infection were first assessed using the SHIRPA battery of tests [33]. Briefly, mice are submitted to 40 quick tests individually, observed and evaluated semi-quantitatively on their performance. The SHIRPA battery was performed in the laboratory animal facility, and results were expressed as a score for each animal in experimental groups. Mice were also assessed for alterations on the spontaneous locomotor activity, using the open-field method. Mice are placed individually in a transparent acrylic cage for 20 min. Their movement was recorded with a video camera and analyzed for total traveled distance, using the software Any-Maze (Stoelting Company). Results are expressed as traveled distance per mouse in a given experimental group.

### In vitro experimental infections

N1E-115 mouse neuroblast cell line was kindly provided by Josiane Piedade (FUNED, Belo Horizonte, Brazil) and maintained in DMEM supplemented with 5 mM HEPES, 10% *v*/*v* SFB, and antibiotics. Primary neuronal cell cultures were obtained from the cortex and striatum areas of C57BL/6 embryos at 15 days post coitum. After dissection, the tissue was digested with trypsin and dissociated. Cells were plated into poli-l-ornitine-coated plaques with Neurobasal medium supplemented with 2nM Glutamax, B-27, and antibiotics (all purchased from Gibco, Thermo Fisher Scientific, USA). Cell cultures were incubated in 37 °C at 5% CO_2_ for 5–7 days until use. Primary mixed glia cultures were obtained by collecting the brains of newborn mice (1–2 days old). Brains were minced and digested in 0.1% *w*/*v* trypsin (Gibco) for 15 min under agitation. Brain homogenates were washed in DMEM twice and plated in cell culture flasks containing DMEM supplemented with 10% FBS. Mixed glial cell cultures were harvested after 9–14 days. Briefly, cell cultures were infected with SLEV at a MOI of 0.1 for 1 h, washed in DMEM and incubated at 37 °C 5% CO_2_ until sample collection (24–96 h post infection). Collected samples included cell culture supernatants for measurement of cytokine levels and viral load. Neuronal death was assessed in primary cultures using the LIVE/DEAD Viability/Cytotoxicity kit for mammalian cells (Molecular probes, Thermo Fisher Scientific, USA) and observed using a FLoid Cell imaging station (Life Technologies).

### Statistical analysis

Results are expressed as mean plus standard error of mean, unless otherwise stated. Raw data were first analyzed for the presence of outliers (GraphPad quickCalcs) and checked for Gaussian distribution. Data sets were compared using ANOVA, followed by Tukey or Sidak post-tests. Differences between survival curves were analyzed using the log-rank test. Results with *P* < 0.05 were considered significant. All data are representative of at least two experiments (*n* = 4 to *n* = 12 replicates or *n* = 7 to *n* = 17 mice).

## Results

### Wild-type adult mice are susceptible to intracranial inoculation of SLEV BeH 355964 in an inoculum-dependent manner

**Fig. 1:**
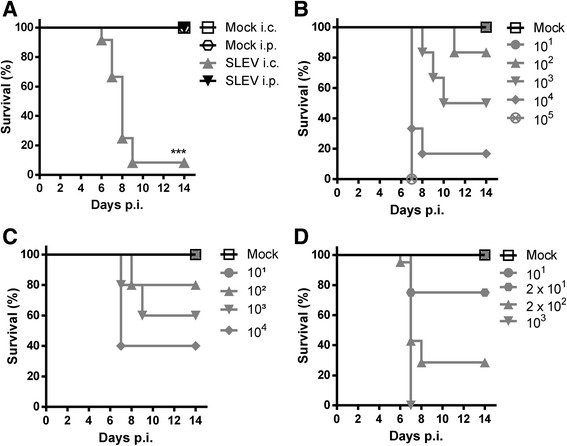
Intracranial inoculation of SLEV BeH 355964 causes disease and mortality in adult wild-type mice in an inoculum-dependent manner. **a** Eight- to 12-week-old BALB/c mice were inoculated intracranially (i.c.) or intraperitoneally (i.p.) with 10^5^ PFU of SLEV BeH 355964 and observed for 14 days post infection (p.i.). **b**, **c**, **d** Eight- to 12-week-old BALB/c (**b**), SV129 (**c**), and C57BL/6 (**d**) were infected i.c. with different inocula of SLEV BeH 355964 and observed for 14 days p.i.. Results are expressed as percentage of survival in each group and are representative of two experiments. Mock = injected with saline. *N* = 6–9 mice. ***P < 0.001 compared to the respective Mock-infected group

Our studies were initiated by the inoculation of SLEV BeH 355964, henceforth referred to as SLEV, in adult (8–12 weeks) wild-type (WT) BALB/c mice. Mice were inoculated with 10^5^ PFU of SLEV via the intraperitoneal (i.p.) or intracranial (i.c.) routes and followed for signs of disease and mortality for 14 days (Fig. 1a). The majority of mice inoculated with SLEV via the i.c. route presented disease signs such as ruffled fur and hunched back at 6 to 7 days after infection (p.i.), which developed into complete paralysis and death. Mice inoculated with SLEV via the i.p. route or injected with saline (Fig. 1a, Mock i.c., Mock i.p.) did not present with disease signs or death. We also performed an experiment in which mice were inoculated with SLEV via intraplantar or subcutaneous (s.c.) routes, in addition to i.p. and i.c. routes already tested. We observed that mice infected by peripheral routes (intraplantar, s.c. and i.p.) did not present disease signs or mortality, whereas mice inoculated with SLEV via the i.c. route manifested disease and mortality at 6-7 days p.i.  (Additional file 1: Figure S1).

To investigate if SLEV-induced mortality was inoculum-dependent, we infected groups of BALB/c (Fig. 1b) with different inocula of SLEV and followed for signs of disease and need for euthanasia. This experiment was performed in parallel with other commonly used laboratory mouse strains (SV129, Fig. 1c) (C57BL/6, Fig. 1d) to test SLEV infection reproducibility. Infected mice of all strains manifested severe disease and death in an inoculum-dependent fashion, although strains had different degrees of susceptibility to SLEV infection. SV129 mice (Fig. 1c) were found to be more resistant to SLEV i.c. infection, followed by BALB/c mice (Fig. 1b) and finally by the more susceptible C57BL/6 mice (Fig. 1d). All mock-infected mice (injected with saline) had no disease signs. In order to minimize variation and inconsistencies, only female mice were used in the following experiments.

Our data indicate that SLEV can cause disease and death in immunocompetent mice when injected directly into the CNS but not when injected systemically. SLEV-induced disease and mortality were inoculum-dependent and reproducible across different commonly used mouse strains.

### SLEV infects and replicates in mice brains

**Fig. 2:**
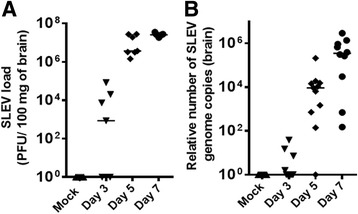
SLEV infects and replicates in the brains of mice. Adult female C57BL/6 mice were inoculated i.c. with 1 LD_100_ of SLEV and euthanized at days 3, 5, and 7 p.i. for brain collection. Viral load in brains was measured by plaque assay (**a**) and by RT-qPCR (**b**). Results are expressed as **a** PFU/100 mg of brain or **b** relative number of SLEV genome copies, including the median for each experiment group. Mock = injected with saline

In order to characterize the disease induced by the i.c. injection of SLEV in mice and ultimately the events leading to death in this model, we inoculated female adult C57BL/6 mice intracranially with 10^3^ PFU of SLEV. This inoculum was selected as it was equivalent to a lethal dose (LD_100_) of SLEV in C57BL/6 mice (Fig. 1d). Mice were euthanized for sample collection at days 3, 5, and 7 after infection, in time points that precede and include the peak of mortality observed in previous experiments. Brain samples were collected, processed, and assessed for viral load by plaque assay (Fig. 2a) and RT-qPCR (Fig. 2b). Our results indicated that the number of plaque forming units (PFU) and SLEV genome copies increase exponentially in the brains of infected mice during the evaluated time points (Fig. 2a, b), which are both undetectable in mock-infected controls. SLEV accumulation in the brain peaked at day 7 p.i., as indicated by both techniques. Thus, our results indicate that, upon i.c. inoculation, SLEV infects and replicates in the mouse brain.

### SLEV infection induces the production of proinflammatory cytokines and interferons in the mouse brain

**Fig. 3:**
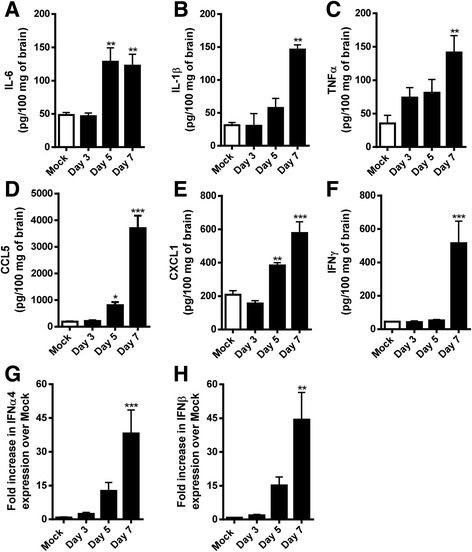
SLEV infection induces the production of cytokines and interferons in the brain of mice. Adult female C57BL/6 mice were inoculated i.c. with 1 LD_100_ of SLEV and euthanized at days 3, 5, and 7 p.i. for brain collection. Levels of the cytokines IL-6 (**a**), IL-1β (**b**), TNF-α (**c**), CCL5 (**d**), CXCL1 (**e**), and IFN-γ (**f**) were quantified by ELISA in 10% *w*/*v* brain homogenates. IFNα4 (**g**) and IFNβ (**h**) levels were quantified by RT-qPCR in RNA extractions from brain samples. The limit of detection of the test is 4–8 pg/mL. Results are expressed as mean plus standard error of the mean (SEM) and are representative of two independent experiments (*N* = 7–14). *P < 0.05, **P < 0.01, ***P < 0.001 compared to the respective Mock-infected group. Mock = injected with saline

SLEV replication in the brain was accompanied by the local production of cytokines, chemokines, and interferons (IFNs). Tissue samples from mock-infected and SLEV-infected mice euthanized at days 3, 5, and 7 p.i. were collected, processed, and assessed by ELISA or by RT-qPCR (Fig. 3). The choice of cytokines was based on the known role of these molecules in the context of neuropathology, either by mediating inflammation (IL-6, IL-1β, TNFα, CCL5, CXCL1) and/or by their association to viral infections (IFNs). All cytokines measured were increased in infected brains as infection progressed to day 7 p.i., when compared to levels observed in the mock-infected group. The cytokines IL-6 (Fig. 3a), CCL5 (Fig. 3d), and CXCL1 (Fig. 3e) were already increased in the brains of SLEV-infected mice at day 5 p.i. In contrast to IL-6, levels of CCL5 and CXCL1 continued to increase and reached peak levels at day 7 p.i.. The cytokines IL-1β (Fig. 3b), TNFα (Fig. 3c), and IFNs (Fig. 3f, g, h) were increased only at day 7 p.i.. In summary, SLEV replication in the brain is associated to the expression of proinflammatory cytokines and IFNs, which reached peak levels at day 7 p.i.

### SLEV infection leads to the recruitment of lymphocytes and neutrophils into the brain of the infected mice

**Fig. 4:**
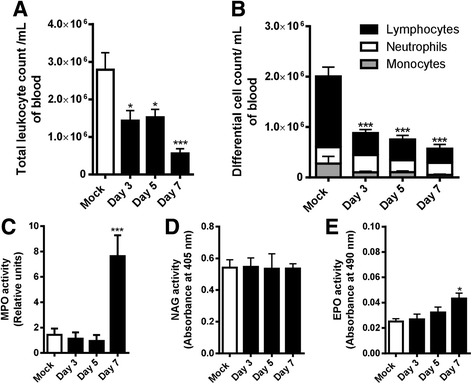
SLEV infection causes leukopenia characterized by lymphopenia in mice and causes the recruitment of granulocytes to the brain. Adult female C57BL/6 mice were inoculated i.c. with 1 LD_100_ of SLEV and euthanized at days 3, 5, and 7 p.i. for collection of blood and brain. Blood samples were used for total (**a**) and differential (**b**) leukocyte counts. Brain samples were processed and used in assays for detection of enzymatic activity of MPO (**c**), NAG (**d**) and EPO (**e**). Results are expressed as mean plus standard error of the mean (SEM) and are representative of two independent experiments (*N* = 6–15). *P < 0.05, ***P < 0.001 compared to the respective Mock-infected group. Mock = injected with saline

The observation that SLEV caused the production of chemokines led us to investigate whether there would be influx of circulating leukocytes into the brain after infection. Blood samples collected from mice inoculated with saline (Mock) or 1 LD_100_ of i.c. inoculated SLEV, at days 3, 5, and 7 p.i., were used for total and differential counting of leukocytes (Fig. 4a, b). Our results showed that SLEV-infected mice present reduced numbers of circulating leukocytes already at day 3 p.i., which is maintained during day 5 and further reduced at day 7 p.i. (Fig. 4a). The differential leukocyte count showed that the leukopenia presented by infected mice was due to lymphopenia, as infected mice had a significant decrease in lymphocyte numbers at all time points evaluated, when compared to uninfected controls (Mock) (Fig. 4b). In addition to leukocyte counts, blood samples were also evaluated for platelet counts and for the hematocrit index. SLEV infection could not alter the numbers of platelets or the hematocrit index, which remained similar to levels presented by saline-injected controls (Mock) (Additional file 2: Figure S2).

To continue our analysis, brain homogenates from infected and control mice were tested for the presence of infiltrating leukocytes through detection of the enzymatic activity of MPO, NAG, and EPO, present in neutrophils, macrophages, and eosinophils, respectively (Fig. 4c, d, e). Our results showed an increase in MPO activity (Fig. 4c) and a minor increase in EPO activity (Fig. 4e) in the brains of SLEV-infected mice at day 7 p.i., relative to the respective Mock controls. No differences were observed for the activity of NAG in mock and SLEV-infected groups (Fig. 4e). These data indicate that neutrophils, and to lesser extent eosinophils, are recruited to SLEV-infected mice brains.

**Fig. 5:**
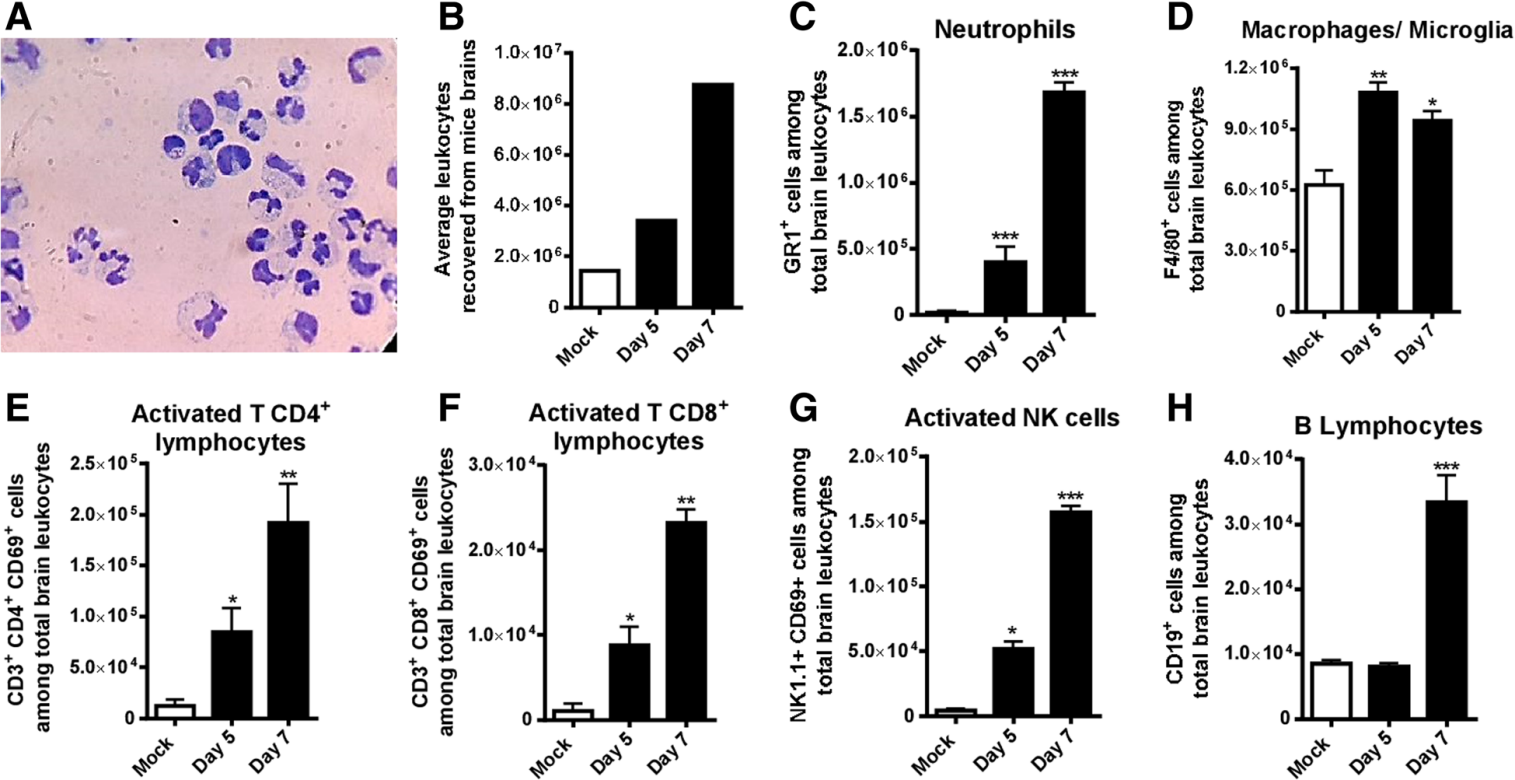
Neutrophils, T lymphocytes, and NK cells are recruited to the brain of SLEV-infected mice. Adult female C57BL/6 mice were inoculated i.c. with 1 LD_100_ of SLEV and euthanized at days 5 and 7 p.i. for collection of brains and extraction of leukocytes. **a** Representative image of Diff-Quik-stained leukocytes recovered from the brains of SLEV-infected mice. **b** Average number of leukocytes recovered from mock and SLEV-infected mice. **c**–**h** Recovered leukocytes were stained with antibodies against leukocyte surface markers and analyzed at a flow cytometer. Results are expressed as mean plus standard error of the mean (SEM) and are representative of one experiment (*N* = 3–6). *P < 0.05, **P < 0.01, ***P < 0.001 compared to the respective Mock-infected group. Mock = injected with saline

In order to confirm our previous results and to investigate the recruitment of lymphocytes in SLEV in vivo infection, flow cytometry experiments were performed (Fig. 5). Groups of mice were injected with saline i.c. or inoculated i.c. with 1 LD_100_ of SLEV. Brains were perfused, collected at days 5 and 7 p.i., and processed for the isolation of leukocytes. Recovered cells were composed mainly of neutrophils and lymphocytes (Fig. 5a) and increased in number as infection progressed (Fig. 5b). Neutrophils were the most prevalent leukocyte population in SLEV infected brains, with an average of 1.5 million cells recruited at day 7 p.i. (Fig. 5c). Macrophages and microglia were both evaluated according to the expression of F4/80 and found to be increased in SLEV-infected groups, although mock-infected mice present a significant amount of F4/80^+^ tissue-resident cells (Fig. 5d). Activated T CD4^+^ (Fig. 5e) and T CD8^+^ lymphocytes (Fig. 5f) were increased in the brains of infected mice at both days 5 and 7 p.i., reaching peak numbers at day 7 p.i.. Activated NK cells were also present in infected brains and also peaked at day 7 after infection (Fig. 5g). B lymphocytes were increased in the brains of infected mice only at day 7 p.i. (Fig. 5h). Mock-injected mice showed no evidence of leukocyte recruitment above basal levels.

Altogether, data presented in this section indicate that SLEV infection leads to lymphopenia and to significant leukocyte recruitment to the brain, composed of neutrophils and lymphocytes. Leukocyte recruitment in SLEV experimental infection follows chemokine production and viral replication.

### SLEV infection does not affect the spleen and does not cause systemic production of cytokines

Next, we examine whether i.c. SLEV infection would also induce systemic inflammation, as it inflamed the brain. Spleen and sera were collected from SLEV-infected and mock-infected mice at different time points p.i. and assessed for viral load and or signs of inflammation (Additional file 3: Figure S3). Spleens of both mock and SLEV-infected mice were negative for SLEV, as assessed by plaque assay (Additional file 3: Figure S3A), and showed no evidence for neutrophil or macrophage recruitment, as measured by the activity of MPO and NAG in tissue samples, respectively (Additional file 3: Figure S3B, C). Levels of CCL5, TNFα, and IFNγ, which are increased in the brains of infected mice, were similar between infected and non-infected mice (mock) in the spleen (Additional file 3: Figure S3D, E, F). Accordingly, CCL5, TNFα, and IFNγ could not be detected in the sera infected or non-infected mice throughout the evaluated time points (Additional file 3: Figure S3G, H, I). In summary, we found no evidence suggesting that SLEV i.c. infection becomes systemic in this model or induces systemic inflammation.

### SLEV infection causes CNS tissue damage and results in behavioral alterations

**Fig. 6:**
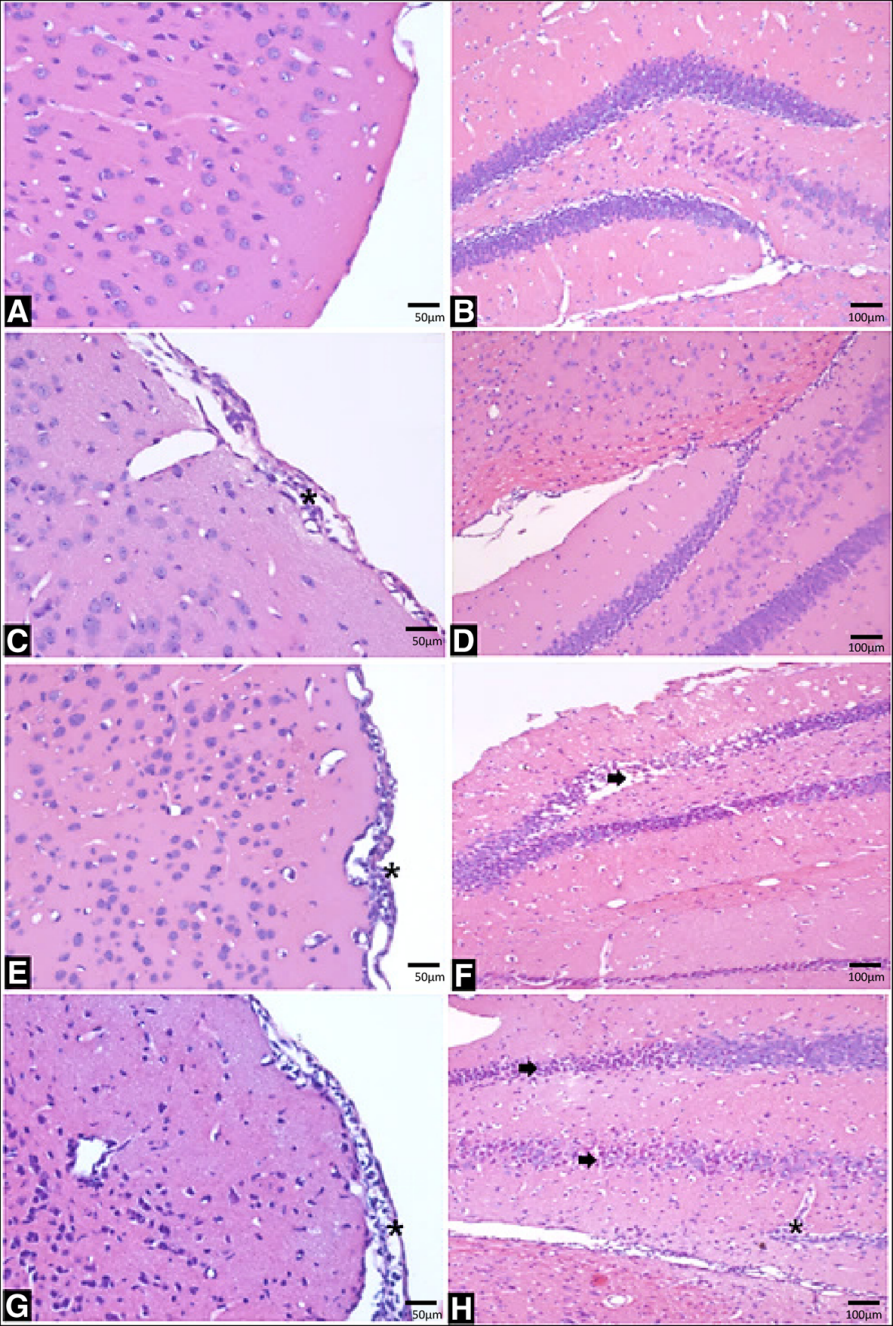
SLEV infection causes brain damage in mice. Adult female C57BL/6 mice were inoculated i.c. with 1 LD_100_ of SLEV and euthanized at days 3, 5, and 7 p.i. for perfusion and collection of brains. Histological sections were stained in H&E and analyzed under an optical microscope. Images of the cerebral cortex/meninges are shown on the *left* (**a**, **c**, **e**, **g**) (magnification ×400) and the hippocampus on the *right* (**b**, **d**, **f**, **h**) (magnification ×200). **a**, **b** Brain histological sections representative of a Mock-infected mouse, followed by images from infected animals at day 3 (**c**, **d**), day 5 (**e**, **f**), and day 7 p.i. (**g**, **h**). Images show the development of meningitis (*asterisk*, **c**, **e**, **g**) and meningoencephalitis with microgliosis (**g**). Tissue damage/degeneration (*arrows*) and (*asterisk*) inflammation in the hippocampus are observed in (**f**, **h**). *N* = 3 mice per group. Mock = injected with saline

**Fig. 7:**
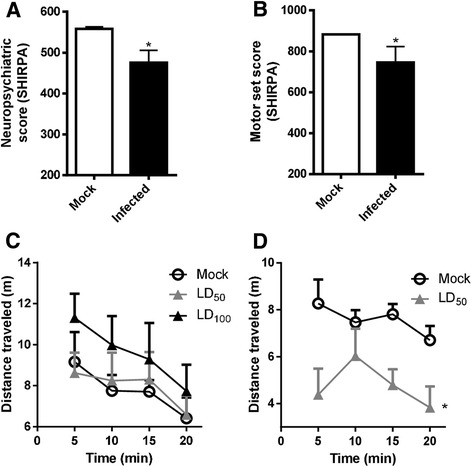
SLEV infection causes neurological alterations in mice. Adult female C57BL/6 mice were inoculated with SLEV or saline i.c. and tested at days 6 or 12 p.i. for neurological alterations. **a**, **b** Mice inoculated with 1LD_100_ of SLEV or saline were submitted to the SHIRPA battery of tests at day 6 p.i., to evaluate neurological scores on neuropsychiatric (**a**) and motor functions (**b**). **c**, **d** Mice inoculated with 1 LD_100_, 1LD_50_, or saline were submitted to the open field test to evaluate mice spontaneous locomotor activity at day 6 or 12 p.i., through measurement of the distance traveled in a defined area for 20 min. Results are expressed as mean plus SEM and are representative of two experiments (*N* = 7–8). Mock = injected with saline

Because i.c. SLEV infection in mice caused brain inflammation and because human infection with SLEV is associated with significant morbidity, we assessed whether SLEV infection induced brain damage and/or functional alterations. Our results showed that SLEV causes progressive pathological alterations in the mouse brain, as compared to normal saline-injected brains (Fig. 6a, b). On day 3, a discrete sign of meningitis is observed (Fig. 6c, asterisk), which evolves to severe meningoencephalitis at days 5 (Fig. 6e) and 7 p.i. (Fig. 6g). The hippocampus was progressively damaged by SLEV infection throughout the evaluated time points (Fig. 6d, f, h, arrows), which may be associated with neuronal death, and presented infiltrating leukocytes at day 7 p.i. (Fig. 6h, asterisk). At day 7 p.i., microgliosis is observed in both cortex and hippocampus (Fig. 6g, h). Histological slides generated in the experiment were also subjected to semi-quantitative analysis (Additional file 4: Figure S4). Our data show that SLEV-infected mice present alterations consistent with meningitis and with damage to the cerebrum and hippocampus (Additional file 4: Figure S4A, B, C). In addition, we found that SLEV infection also caused significant alterations in the brainstem (Additional file 4: Figure S4D).

In order to characterize the behavioral alterations caused by SLEV infection, we used the SHIRPA battery of tests and the open-field test (Fig. 7). For the SHIRPA test, mock-infected and SLEV-infected mice at day 6 p.i. were subjected to a series of quick tests, to measure aspects of murine neurological function. The time point chosen (day 6 p.i.) is due to the fact that infected mice at day 7 p.i. are mostly incapacitated and thus, not able to perform any tests. Our results showed that SLEV-infected mice present a reduction in neuropsychiatric (Fig. 7a) and motor function (Fig. 7b) scores when compared to mock-infected mice, indicating that these neurological functions are affected by SLEV infection.

The open-field test measures the spontaneous movement of a mouse in a defined area. For this experiment, groups of mice were inoculated with saline (Mock), 1 LD_100_ or 1 LD_50_ of SLEV i.c., to compare mice that receive lethal or sublethal inocula of SLEV, and to study mice that survive the infection among those receiving a sublethal inoculum. We observed that at day 6 p.i., mice from control and infected groups traveled similar distances (Fig. 7c), indicating that these mice had the same rate of spontaneous movement. At day 12 p.i., 4–5 days after the onset of severe disease/mortality, surviving mice present reduced spontaneous movement, as observed by reduction in the distances traveled during the open-field test in comparison to the mock control group (Fig. 7d).

In summary, we observed that SLEV i.c. infection causes progressive brain damage in mice, likely including neuronal loss, that is characterized by meningoencephalitis. The extent of tissue damage correlates SLEV load and inflammation, suggesting that these processes are associated. The onset of experimental St. Louis encephalitis is preceded and followed by neurological alterations, especially in motor function.

### SLEV is pathogenic to neurons and glial cells in vitro

**Fig. 8:**
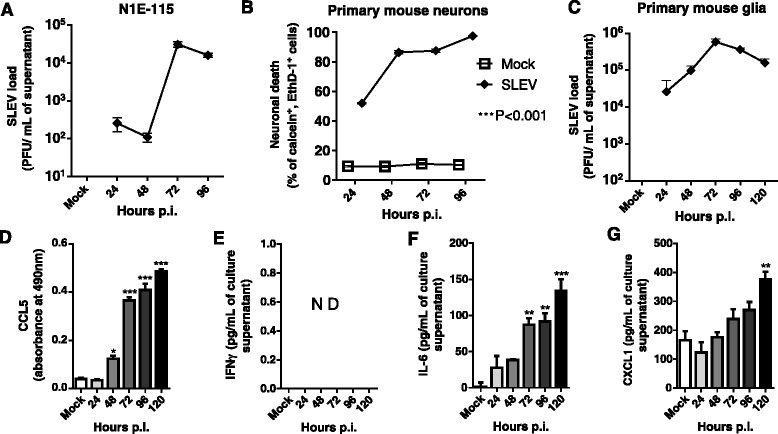
SLEV is pathogenic to murine neurons and glial cells in vitro. Mouse brain cell cultures were prepared and infected with SLEV at a MOI of 0.1, or incubated with medium (Mock). Samples were collected and/or cell cultures were assessed at 24–120 h p.i. **a** SLEV load in the supernatant of N1E-115 murine neuroblast cell cultures, measured by plaque assay. **b** Percentage of dead neurons in a primary murine neuron culture infected with SLEV, measured by the percentage of positive cells for calcein and Ethidium homodimer-1 (EthD-1) staining. **c** SLEV load in the supernatant of primary murine mixed glial cell cultures, measured by plaque assay. **d**–**g** Primary murine glial cells were assessed for the release of proinflammatory cytokines CCL5 (**d**), IFNγ (**e**), IL-6 (**f**), and CXCL1 (**g**) into the culture supernatant, by ELISA. Results are expressed as mean plus SEM and are representative of two experiments (*N* = 4–12). Mock = not infected, incubated with medium. *ND* not detectable

In order to identify the main cell types infected by SLEV, we performed a series of in vitro infections of cells representing the main cell populations present in the CNS: neurons and glia (Fig. 8). The murine neuroblast cell line N1E-115 was first tested and found to be permissive to SLEV infection and replication (Fig. 8a). When infected with SLEV at a multiplicity of infection (MOI) of 0.1, N1E-115 cells released infective SLEV in the culture supernatant at all time points evaluated (24–96 h p.i.), reaching peak levels at 72 h p.i.. We next performed a primary culture of murine neurons isolated from C57BL/6 mice, which were infected with SLEV at a MOI of 0.1 and assessed for neuronal death (Fig. 8b). We observed that primary murine neurons are very susceptible to SLEV infection, as SLEV-infected cultures presented 50% cell death already at 24 h p.i.. Neuronal death increased to 80% at 48 and 72 h p.i. and resulted in complete death at 96 h p.i., when compared to mock-infected controls, which received culture medium.

Primary glial cultures were obtained from newborn C57BL/6 mice and consisted in a confluent mixed culture of astrocytes, oligodendrocytes, and microglia. Cultures were again infected with SLEV at a MOI of 0.1 and maintained for up to 120 h for supernatant collection (Fig. 8c, d). Samples were assessed for their viral content and results showed that SLEV replicates in glial cells, reaching peak levels in cultures at 72 h p.i. (Fig. 8c). SLEV replication was associated to the release of proinflammatory cytokines in the culture supernatant (Fig. 8d–g). Cytokines CCL5, IL-6, and CXCL1 were detected in glia culture supernatants and increased along the time points evaluated in SLEV-infected cultures. CCL5 was detected earlier in glial cultures, increasing in infected cultures at 48 h p.i. in comparison to Mock, and increasing further at 72, 96, and 120 h p.i. (Fig. 8d). IL-6 was increased in cell cultures at 72 and 96 h p.i. and reached peak levels at 120 h p.i. (Fig. 8f). CXCL1 was increased in comparison to Mock controls only at 120 h p.i. (Fig. 8g). Notably, IFNγ (Fig. 8e) was not detected in control or SLEV-infected cultures at any of the evaluated time points.

In conclusion, we suggest that SLEV is able to infect and replicate in cultures of both neuronal and glial cells of mice. In vitro SLEV infection is pathogenic, capable of inducing neuronal cell death and cytokine release by glial cells, which correlates with SLEV pathogenicity in vivo and suggests that SLEV may interact with more than one cell type in the brain.

## Discussion

In this manuscript, we presented a robust model of St. Louis encephalitis in mice, characterized by viral replication, inflammation, brain damage, neurological alterations, and death. Importantly, these are main characteristics of severe disease caused by SLEV in humans [13, 18, 30, 34] that are reproduced in this experimental model. In addition, the observation that SLEV infects and replicates in murine neuronal cells correlates with the presence of SLEV in human neurons [35].

Up to now, mouse models of St. Louis encephalitis have focused on infection indices and mortality rates [25, 26, 28] and lacked insights into the contribution of inflammation to severe disease pathogenesis. Inflammation is a major feature of flaviviral disease, as extensively demonstrated for dengue, West Nile, and Japanese encephalitis [36–41]. In our model, the majority of disease parameters evaluated reach peak levels at day 7 p.i. and immediately preceded the manifestation of severe disease and death. Altogether, we suggest that day 7 p.i. is the peak of infection/disease in this model.

The SLEV strain BeH 355964 was chosen to establish this in vivo model because of key features: SLEV BeH 355964 was originally isolated from a symptomatic patient in Brazil [30], and thus it is associated to disease occurrence in humans; SLEV BeH 355964 is a reference SLEV strain used for research purposes [11, 31, 42, 43], and its entire genome sequence was recently published [5]. Also, adult immunocompetent mice were chosen because their immune and inflammatory responses are intact, contrary to the biased response of knockout or young mice, which are known to be more susceptible to other routes of virus inoculation [15, 25, 27, 44]. Thus, we designed our experimental SLEV model to prioritize the study of the host immunological response to a clinically relevant virus isolate. Unfortunately, our model was limited by the fact that BeH 355964 was unable to cause disease in adult immunocompetent mice when injected peripherally. By injecting virus i.c., we skip initial phases of infection at the skin and lymphoid tissues, which are believed to precede flavivirus invasion of the CNS [45]. Therefore, we consider our animal model to better represent SLEV-induced severe disease, where SLEV has already invaded the CNS, and causes meningoencephalitis.

We observed by different techniques that i.c. SLEV inoculation in mice results in acute brain inflammation, corroborating previous reports [22, 24]. We suggest that SLEV replication in the brain triggers the local production of proinflammatory cytokines that ultimately causes SLEV-induced meningoencephalitis. We have performed preliminary experiments using mice deficient in the platelet-activating factor receptor (PAFR^−/−^) or deficient in the complement C5a receptor (C5aR^−/−^) and found that these molecules were not involved in the pathogenesis of experimental St. Louis encephalitis. Among the cytokines evaluated in this study, the chemokines CCL5 and CXCL1 (Fig. 3) are known to mediate the recruitment of lymphocytes [46] and neutrophils [47] and are likely to mediate the infiltration these leukocyte populations into the SLEV-infected brain [45]. Clinical or post mortem studies in individuals diagnosed with St. Louis encephalitis frequently showed meningitis associated with lymphocytic and granulocytic infiltration, as well as pleocytosis [13, 34, 48]. The observation that glial cells are activated during SLEV infection and release proinflammatory cytokines in vitro (shown in Fig. 8) is suggestive that these cells may initiate the inflammatory response to SLEV in vivo [43]. Moreover, we observed an increase in a F4/80^+^ population, presumably microglia, and identified microgliosis in histological analysis of SLEV-infected brains which support the involvement of microglia in the infection.

IFNγ was not expressed by SLEV-infected glial cells in vitro. However, IFNγ levels increase in mice brains at the peak of SLEV infection, together with cytokines that were found to be produced by glia, such as CCL5 and IL-6. We suggest that recruited leukocyte populations could be source of IFNγ in vivo, most likely NK and T lymphocytes. These lymphocyte populations are activated and abundant in the brains of infected mice at day 7 p.i. and are known to produce IFNγ in response to flaviviral infection [49]. Moreover, IFNγ was pathogenic in a murine model of Japanese encephalitis by causing blood-brain barrier disruption [40]. The correlation of IFNγ expression in SLEV-infected brains and the onset of severe neurological disease and mortality indicate that IFNγ could be involved in the pathogenesis of experimental St. Louis encephalitis, but this contention clearly deserves further experimentation.

## Conclusions

The association of SLEV infection with a major inflammatory response in the brain, and to severe neurological damage and dysfunction, suggests that inflammation plays a major role in St. Louis encephalitis pathogenesis. More studies are necessary to fully elucidate the mechanisms by which SLEV causes this pathogenic host response to infection in the CNS, resulting in tissue damage and in death. We suggest that therapeutic strategies to reduce CNS inflammation may be beneficial in the context of St. Louis encephalitis.

## Additional files

Additional file 1: Figure S1. Adult immunocompetent mice are resistant to SLEV when inoculated through peripheral routes. Eight- to 12-week-old female C57BL/6 mice were inoculated with 10^3^ PFU of SLEV BeH 355964 through different routes (intraperitoneal, intraplantar, subcutaneous, and intracranial) and observed for 14 days post infection. Results are expressed as percentage of survival in each group and is representative of one experiment. *N* = 5 mice. (TIF 40 kb)

Additional file 2: Figure S2. Experimental SLEV infection does not change platelet counts or the hematocrit index. Adult female C57BL/6 mice were inoculated i.c. with 1 LD_100_ of SLEV and euthanized at days 3, 5, and 7 p.i. for blood collection. Platelet counts (A) and the hematocrit index (B) were quantified in heparinized samples. Results are expressed as mean plus SEM and are representative of one experiment (*N* = 3–8). Mock = injected with saline. (TIFF 120 kb)

Additional file 3: Figure S3. Intracranial SLEV infection does not affect the spleen or causes systemic inflammation. Adult female C57BL/6 mice were inoculated i.c. with 1 LD_100_ of SLEV and euthanized at days 3, 5, and 7 p.i. for collection of spleens and sera. Spleen samples were processed and assessed for SLEV load (A) by plaque assay and enzymatic activity of MPO (B) and NAG (C). Levels of the cytokines CCL5, TNFα, and IFNγ were measured in spleen (D, E, F) and serum samples (G, H, I). Results are expressed as dot plot or mean plus SEM and are representative of two experiments (*N* = 6–12). Mock = injected with saline. ND = not detectable. (TIFF 360 kb)

Additional file 4: Figure S4. Histopathological alterations in SLEV-infected mice are quantifiable in the meninges, brain, hippocampus, and brainstem. Adult female C57BL/6 mice were inoculated i.c. with 1 LD_100_ of SLEV and euthanized at days 3, 5, and 7 p.i. for collection of brains for a histological semi-quantitative analysis. Slides were scored on up to four points, with four corresponding to maximum tissue damage. Scores were set based on the histological aspect of samples from the mock-infected group. Brain regions analyzed included the meninges (A), the cerebrum (B), the hippocampus (C) and the brainstem (D). Results are expressed as dot-plot and the median for each experiment group. *P < 0.05, **P < 0.01 compared to the respective day 3 p.i. group. Mock = injected with saline. (TIFF 122 kb)

## Abbreviations

ANOVA: Analysis of variance
CEUA: Internal committee of ethics on animal use
CNS: Central nervous system
EPO: Eosinophil peroxydase
FBS: Fetal bovine serum
i.c.: Intracranial
i.p.: Intraperitoneal
IFN: Interferon
JEV: Japanese encephalitis virus
LD_50/100_: Lethal dose for 50%/100% subjects
MOI: Multiplicity of infection
MPO: Mieloperoxydase
NAG: *N*-Acetyl-β-d-glucosaminidase
p.i.: Post infection
PFU: Plaque forming units
RT-qPCR: Reverse transcriptase quantitative PCR
SEM: Standard error of the mean
SHIRPA: SmithKline Beecham, Harwell, Imperial College, Royal London Hospital, phenotype assessment
SLEV: St. Louis encephalitis virus
St.: Saint
UFMG: Universidade Federal de Minas Gerais
WNV: West Nile virus
WT: Wild type
ZIKV: Zika virus
