# Supplementary figures and images for: Development of a model of Saint Louis encephalitis infection and disease in mice
Source: J Neuroinflammation. 2017 Mar 22;14:61. doi: 10.1186/s12974-017-0837-2 (PMC5361699; doi:10.1186/s12974-017-0837-2)

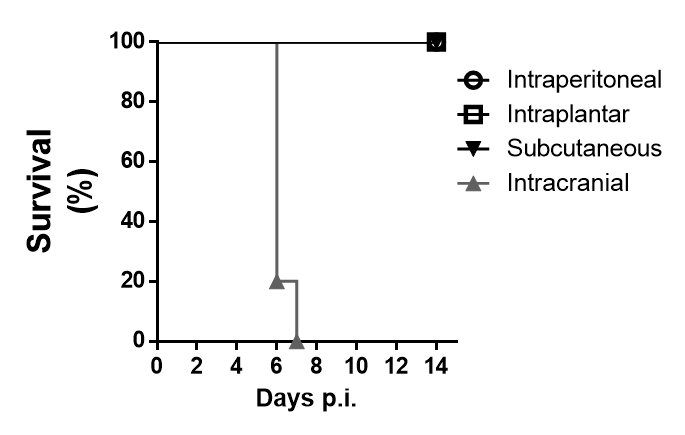

Supplement: Supplementary file 1 — Adult immunocompetent mice are resistant to SLEV when inoculated through peripheral routes. Eight- to 12-week-old female C57BL/6 mice were inoculated with 103 PFU of SLEV BeH 355964 through different routes (intraperitoneal, intraplantar, subcutaneous, and intracranial) and observed for 14 days post infection. Results are expressed as percentage of survival in each group and is representative of one experiment. N = 5 mice. (TIF 40 kb) [file 12974_2017_837_MOESM1_ESM.tif]

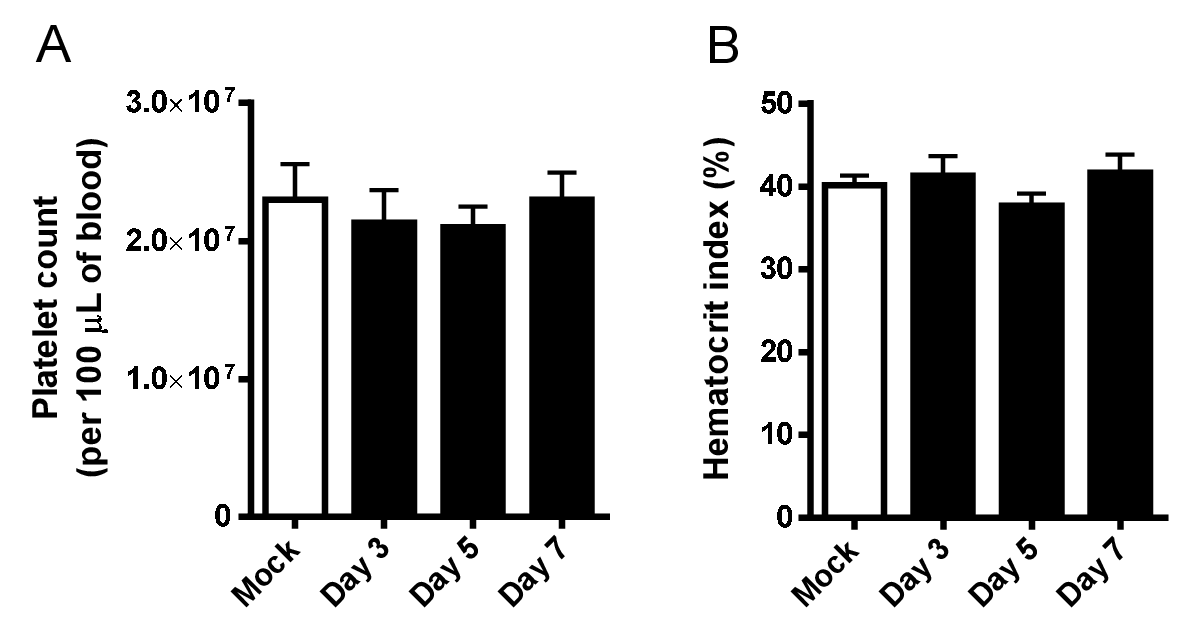

Supplement: Supplementary file 2 — Experimental SLEV infection does not change platelet counts or the hematocrit index. Adult female C57BL/6 mice were inoculated i.c. with 1 LD100 of SLEV and euthanized at days 3, 5, and 7 p.i. for blood collection. Platelet counts (A) and the hematocrit index (B) were quantified in heparinized samples. Results are expressed as mean plus SEM and are representative of one experiment (N = 3–8). Mock = injected with saline. (TIFF 120 kb) [file 12974_2017_837_MOESM2_ESM.tiff]

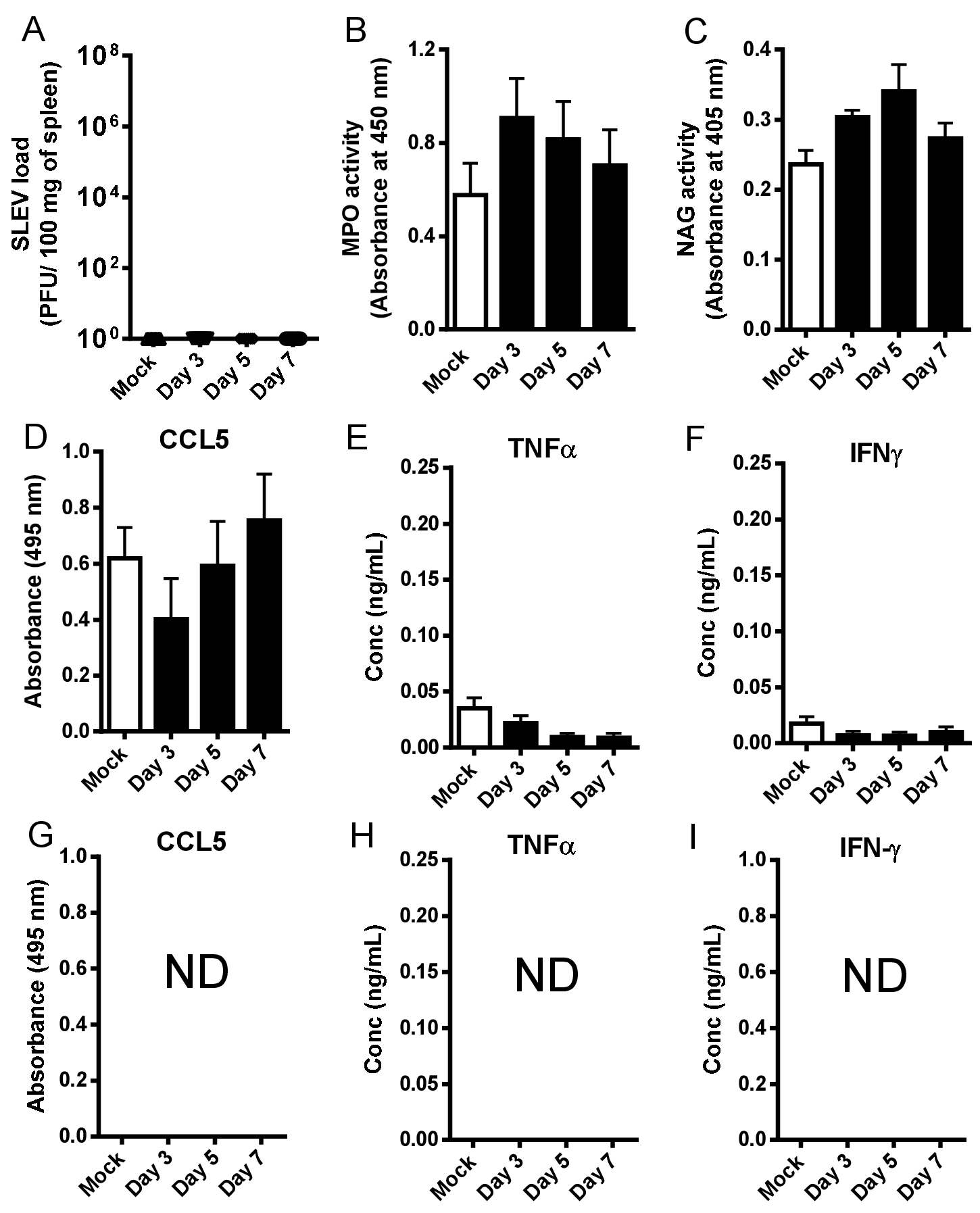

Supplement: Supplementary file 3 — Intracranial SLEV infection does not affect the spleen or causes systemic inflammation. Adult female C57BL/6 mice were inoculated i.c. with 1 LD100 of SLEV and euthanized at days 3, 5, and 7 p.i. for collection of spleens and sera. Spleen samples were processed and assessed for SLEV load (A) by plaque assay and enzymatic activity of MPO (B) and NAG (C). Levels of the cytokines CCL5, TNFα, and IFNγ were measured in spleen (D, E, F) and serum samples (G, H, I). Results are expressed as dot plot or mean plus SEM and are representative of two experiments (N = 6–12). Mock = injected with saline. ND = not detectable. (TIFF 360 kb) [file 12974_2017_837_MOESM3_ESM.tiff]

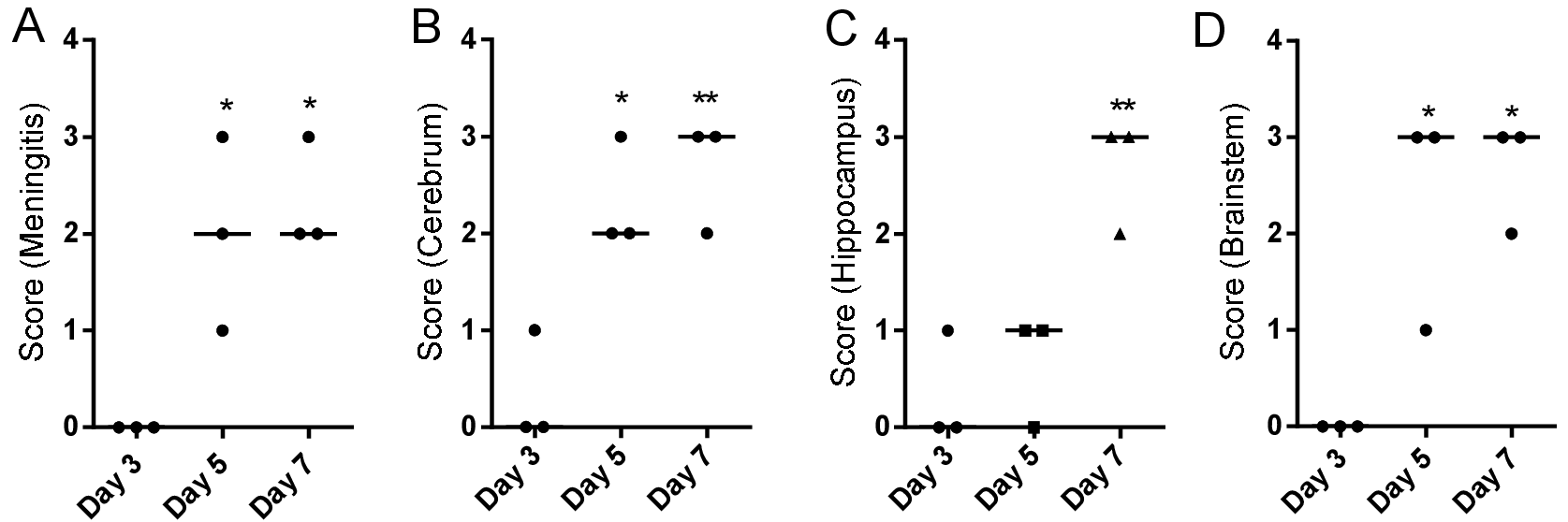

Supplement: Supplementary file 4 — Histopathological alterations in SLEV-infected mice are quantifiable in the meninges, brain, hippocampus, and brainstem. Adult female C57BL/6 mice were inoculated i.c. with 1 LD100 of SLEV and euthanized at days 3, 5, and 7 p.i. for collection of brains for a histological semi-quantitative analysis. Slides were scored on up to four points, with four corresponding to maximum tissue damage. Scores were set based on the histological aspect of samples from the mock-infected group. Brain regions analyzed included the meninges (A), the cerebrum (B), the hippocampus (C) and the brainstem (D). Results are expressed as dot-plot and the median for each experiment group. *P < 0.05, **P < 0.01 compared to the respective day 3 p.i. group. Mock = injected with saline. (TIFF 122 kb) [file 12974_2017_837_MOESM4_ESM.tiff]
